# Adjuvant chemotherapy and survival outcomes in rectal cancer patients with good response (ypT0-2N0) after neoadjuvant chemoradiotherapy and surgery: A retrospective nationwide analysis

**DOI:** 10.3389/fonc.2022.1087778

**Published:** 2022-12-16

**Authors:** Yu-Hsuan Kuo, Yun-Tzu Lin, Chung-Han Ho, Chia-Lin Chou, Li-Chin Cheng, Chia-Jen Tsai, Wei-Ju Hong, Yi-Chen Chen, Ching-Chieh Yang

**Affiliations:** ^1^ Division of Hematology and Oncology, Department of Internal Medicine, Chi Mei Medical Center, Tainan, Taiwan; ^2^ Department of Cosmetic Science, Chia-Nan University of Pharmacy and Science, Tainan, Taiwan; ^3^ Department of Medical Research, Chi Mei Medical Center, Tainan, Taiwan; ^4^ Department of Information Management, Southern Taiwan University of Science and Technology, Tainan, Taiwan; ^5^ Division of Colorectal Surgery, Department of Surgery, Chi Mei Medical Center, Tainan, Taiwan; ^6^ Department of Medical Laboratory Science and Biotechnology, Chung Hwa University of Medical Technology, Tainan, Taiwan; ^7^ Department of Radiation Oncology, Chi Mei Medical Center, Tainan, Taiwan; ^8^ Department of Pharmacy, Chia-Nan University of Pharmacy and Science, Tainan, Taiwan

**Keywords:** rectal cancer, neoadjuvant chemoradiotherapy, surgery, adjuvant chemotherapy, survival

## Abstract

**Background:**

For rectal cancer, it remains unclear how to incorporate tumor response to neoadjuvant chemoradiotherapy (nCRT) when deciding whether to give adjuvant chemotherapy. In this study, we aim to determinate the survival benefit of adjuvant chemotherapy for rectal cancer patients with good response (ypT0-2N0) after nCRT and surgery.

**Methods:**

The study cohort included 720 rectal cancer patients who had good response (ypT0-2N0) after nCRT and surgery, who did or did not receive adjuvant chemotherapy between January 2007 and December 2017, from the Taiwan Cancer Registry and National Health Insurance Research database. The Kaplan–Meier method, log-rank tests, and Cox regression analysis were performed to investigate the effect of adjuvant chemotherapy on 5-year overall survival (OS) and disease-free survival (DFS).

**Results:**

Of 720 patients, 368 (51.1%) received adjuvant chemotherapy and 352 (48.9%) did not. Patients who received adjuvant chemotherapy were more likely to be female, younger (≤ 65), with advanced clinical T (3-4)/N (1-2) classification and ypT2 classification. No significant difference in 5-year OS (*p*=0.681) or DFS (*p*=0.942) were observed by receipt of adjuvant chemotherapy or not. Multivariable analysis revealed adjuvant chemotherapy was not associated with better OS (adjusted hazard ratio [aHR], 1.03; 95% Confidence Interval [CI], 0.88-1.21) or DFS (aHR, 1.05; 95% CI, 0.89-1.24). Stratified analysis for OS and DFS found no significant protective effect in the use of adjuvant chemotherapy, even for those with advanced clinical T or N classification.

**Conclusion:**

Adjuvant chemotherapy may be omitted in rectal cancer patients with good response (ypT0-2N0) after nCRT and surgery.

## Background

Although mortality has steadily decreased since 1990, colorectal cancer remains one of the most frequent cancer-related death in the US (1). In addition, the incidence of colorectal cancer under the age of 50 increased from 1992 to 2012 at a rate of 2.1% annually, and continues to rise (2). According to the Taiwan Cancer Registry (TCR) database in recent two decades, male and female young-onset rectal cancer incidence rates rose from 4.0 to 8.3 and 3.8 to 6.4 per 100,000 (3).

Although radical resection is the cornerstone of management in rectal cancer, radiotherapy and chemotherapy has emerged as an important component of curative therapy, because local recurrence is more common in those types than with colon primaries (4, 5). Treatment for locally advanced rectal cancer (T3-4N0 or T1-4N1-2) consists of neoadjuvant chemoradiotherapy (nCRT) followed by total mesorectal excision and adjuvant chemotherapy with fluorouracil and oxaliplatin. The use of nCRT promotes greater sphincter preservation and facilitates tumor downstaging (6, 7). Most importantly, about 15% of these patients have a pathologic complete response (defined as ypT0N0), which is associated with an excellent long-term survival outcome (8, 9). A further 20% of patients downstage to ypT1/T2N0 (10). However, up to a third of contemporary patients who undergo surgical resection of rectal cancer patients still ultimately develop metastatic disease (11). Adjuvant chemotherapy after nCRT and resection has thus been proposed as a potential method of alleviating micrometastasis, hence reducing recurrence and enhancing survival. Currently, the use of adjuvant chemotherapy in rectal cancer patients remains controversial (12–14). National Comprehensive Cancer Network (NCCN) guidelines state that pre-treatment staging, not surgical pathology, should be used to guide decisions for adjuvant chemotherapy. It is unclear how to incorporate tumor response to nCRT when deciding to provide adjuvant chemotherapy; this uncertainty is reflected in the poor compliance with NCCN guidelines for adjuvant chemotherapy administration (15).

In this study, we sought to address the impact of adjuvant chemotherapy on survival in rectal cancer patients with good response (ypT0-2N0) after nCRT and surgery, by conducting an analysis from a large cohort of patients from the TCR and the National Health Insurance Research Database (NHIRD).

## Materials and methods

### Ethics approval and informed consent

The study was approved by the Ethics Committee of the Institutional Review Board of Chi Mei Medical Center (IRB: 10707–012). Informed consent was not obtained because the IRB waived the need for individual informed consent, as no personally identifiable information were used. This study had a non-interventional retrospective design, no human subjects used and all data were analyzed anonymously.

### Data source and study cohort

**Figure 1:**
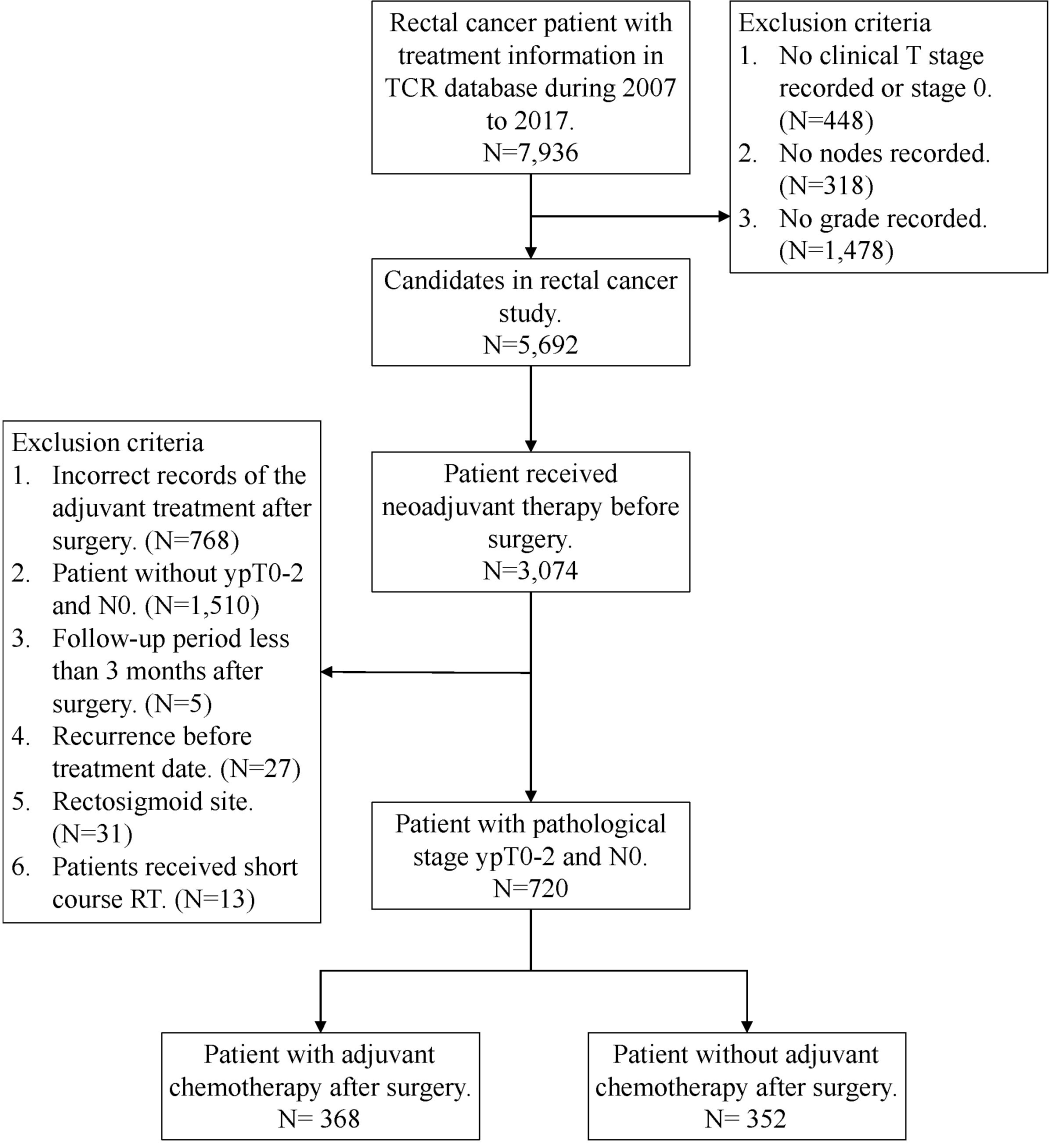
Flow chart.

We obtained data from the TCR and NHIRD, which cover more than 95% of the cancer cases in Taiwan. The TCR also has documented excellent data quality and completeness (16). The rectal cancer patients who underwent nCRT and surgical resection with or without adjuvant chemotherapy between January 2007 and December 2017 were included. Follow-up was completed on December 31, 2018. Exclusion criteria included: 1) a history of cancer or metastatic disease; 2) no clear coding on follow-up and treatment; or 3) patients received short course radiation. Rectal cancer diagnosis were defined by the International Classification of Disease for Oncology, third edition (ICD-O-3) codes for location: rectum (code C20.9); and histologic type: adenocarcinoma (codes 8140, 8210, 8261, and 8263), mucinous adenocarcinoma (code 8480), or signet ring cell carcinoma (code 8490). These patients were all staged according to the 7^th^ edition American Joint Committee on Cancer (AJCC) classification system. The variables from the TCR database used for analysis included age, gender, histology type, grade, stage, margin status, lymph node yield, comorbid conditions and cancer-related treatment. Here, Charlson Comorbidity Index (CCI) score were used to grade the severity of comorbid conditions (17). Finally, a total of 720 rectal cancer patients with good response “ ypT0-2N0 “ after nCRT and surgery were extracted (**Figure 1**).

### Statistical analysis

In this study, all statistical analyses were performed using SAS 9.4 for Windows (SAS Institute, Inc., Cary, NC, USA) and Kaplan-Meier curves were plotted using STATA (version 12; Stata Corp., College Station, TX, USA). A *p* value ≤ 0.05 was considered statistically significant. The distribution difference between ypT0-2N0 rectal cancer patients treated with and without adjuvant chemotherapy was estimated using Pearson’s chi-square test for categorical variables and the Wilcoxon rank sum test for continuous variables. The primary endpoints were the 5-year overall survival (OS) and disease-free survival (DFS) rates and were calculated *via* Kaplan-Meier method, comparing by log-rank statistics. The risk was presented as hazard ratios (HRs) with 95% confidence intervals (CIs) and calculated using the Cox proportional hazards model for factors associated with survival. We also performed stratified survival analyses for important prognostic characteristics such as age, cT/cN/ypN classification.

## Results

Between 2007 and 2017, a total of 720 rectal cancer patients were selected for analysis, including 368 patients receiving adjuvant chemotherapy and 352 patients without adjuvant chemotherapy (**Figure 1**). Baseline clinicopathological characteristics are summarized in **Table 1**. Among these patients, 498 were male (69.2%) and 222 were female (30.8%). The mean age at diagnosis was 61 ± 11 years, and the median (Q1-Q3) follow‐up time was 4.22 years. The information of neoadjuvant treatment is summarized in **Supplementary Table 1**. The median (Q1-Q3) total dose of radiotherapy was 50.4 Gy in 27 fractions. Concurrent, neoadjuvant chemotherapy regimens included fluorouracil (5-FU), leucovorin, capecitabine, oxaliplatin and UFUR. Patients who received adjuvant chemotherapy were more likely to be female, younger (≤ 65), with advanced clinical T (3-4)/N (1-2) classification and ypT2 classification. The median (Q1-Q3) timing of adjuvant chemotherapy started after operation were 36 days. Adjuvant chemotherapy regimens used as follows: Leucovorin, Fluorouracil (5-FU), UFUR, oxaliplatin and capecitabine. Most patients (88.59%) received combined regimens instead of mono-therapy.

**Table 1:** Clinicopathological information of rectal cancer patients with good response (ypT0-2N0) after nCRT and surgery, n=720.

Variable	Adjuvant chemotherapy		*P*-value^b^
	Without	With	
Overall	N=352	N=368	
Age group			
≦65	200 (56.82)	246 (66.85)	0.006
>65	152 (43.18)	122 (33.15)	
Gender			
Male	258 (73.30)	240 (65.22)	0.019
Female	94 (26.70)	128 (34.78)	
Morphology			
Adenocarcinoma	349 (99.15)	364 (98.91)	1.000
Mucinous/signet	3 (0.85)	4 (1.09)	
Grade			
Well	319 (90.63)	336 (91.30)	0.751
Others	33 (9.38)	32 (8.70)	
cT classification			
1-2	78 (22.16)	62 (16.85)	0.072
3-4	274 (77.84)	306 (83.15)	
cN classification			
0	140 (39.77)	122 (33.15)	0.065
1-2	212 (60.23)	246 (66.85)	
ypT classification			
0	155 (44.03)	115 (31.25)	0.002
1	34 (9.66)	46 (12.50)	
2	163 (46.31)	207 (56.25)	
LN yield			
<12	172 (48.86)	167 (45.38)	0.349
≧12	180 (51.14)	201 (54.62)	
Surgery type			
LAR	246 (69.89)	255 (69.29)	0.966
APR	53 (15.06)	58 (15.76)	
Others	53 (15.06)	55 (14.95)	
CCI score			
0	216 (61.36)	231 (62.77)	0.739
1	83 (23.58)	89 (24.18)	
≧2	53 (15.06)	48 (13.04)	
Follow-up period^a^, year			
Median (Q1-Q3)	4.22 (2.48-5.00)	4.24 (2.24-5.00)	0.956

a. The end of study was 2018.12.31 and the study period was defined as the patients were examined 5 years after 3 months of surgery.

b. P-value was derived from Pearson’s Chi-square test for categorical variable and the Wilcoxon rank sum test for the difference in the median of follow-up period between two groups.

nCRT, neoadjuvant chemoradiotherapy; cT, clinical tumor; cN, clinical nodal; ypT, pathological tumor; LN, lymph node; LAR, low anterior resection; APR, abdominoperineal resection; CCI, Charlson Comorbidity Index.

**Figure 2:**
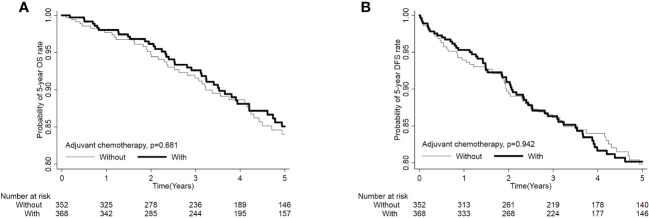
Probability of overall survival **(A)** and disease-free survival **(B)** in rectal cancer patients with good response (ypT0-2N0) after nCRT and surgery, n=720.

Kaplan-Meier survival curves were generated to compare the 5-year OS and DFS rates by receipt of adjuvant chemotherapy or not. As presented in **Figure 2**, there was no significant difference in 5-year OS (*p*=0.681) or DFS (*p*=0.942) between those who received adjuvant chemotherapy and those who did not. After adjustment for confounders (**Table 2**), multivariable analysis indicated that adjuvant chemotherapy was not significantly associated with better 5-year OS (adjusted hazard ratio [aHR], 1.03; 95% Confidence Interval [CI], 0.88-1.21) or DFS (aHR, 1.05; 95% CI, 0.89-1.224). Further stratified analysis for 5-year OS and DFS found no significant protective role in the use of adjuvant chemotherapy for these “good response” rectal cancer patients, even for those with advanced cT or cN classification (**Table 2**). In this study, we selected ypT1-2N0 patients without clinical metastases and there are some early stage (cT1-2N0) included into our analysis. As shown in **Table 3**, comparisons by different clinical and pathological T and N classifications revealed that the use of adjuvant chemotherapy provided limited survival benefit compared to not using it. Regarding to different adjuvant chemotherapy regimens, the related 5-year OS and DFS rate were presented in **Table 4**.

**Table 2:** Overall and stratified analysis of the use of adjuvant chemotherapy for 5-year OS and DFS in rectal cancer patients with good response (ypT0-2N0) after nCRT and surgery, n=720.

With adjuvant chemotherapy vs without	5-year OS				5-year DFS			
	Crude hazard ratio (95% C.I.)	P-value	Adjusted hazard ratio^c^ (95% C.I.)	P-value	Crude hazard ratio (95% C.I.)	P-value	Adjusted hazard ratio^c^ (95% C.I.)	P-value
Overall	1.00 (0.86-1.17)	0.988	1.03 (0.88-1.21)	0.711	1.01 (0.87-1.19)	0.862	1.05 (0.89-1.24)	0.556
Age group								
≦65	0.98 (0.81-1.19)	0.820	1.01 (0.83-1.24)	0.909	1.00 (0.82-1.21)	0.959	1.03 (0.84-1.27)	0.760
>65	1.03 (0.79-1.35)	0.806	1.09 (0.83-1.44)	0.529	1.05 (0.80-1.37)	0.750	1.11 (0.84-1.48)	0.463
cT classification								
1-2	1.04 (0.72-1.48)	0.846	0.99 (0.66-1.47)	0.944	1.07 (0.74-1.55)	0.730	1.06 (0.70-1.59)	0.798
3-4	0.99 (0.83-1.18)	0.914	1.04 (0.87-1.24)	0.693	1.00 (0.84-1.20)	0.973	1.05 (0.88-1.26)	0.592
cN classification								
0	0.84 (0.65-1.10)	0.199	0.83 (0.63-1.10)	0.195	0.83 (0.63-1.09)	0.183	0.82 (0.62-1.10)	0.184
1-2	1.08 (0.89-1.30)	0.464	1.15 (0.94-1.40)	0.184	1.11 (0.91-1.35)	0.299	1.18 (0.96-1.45)	0.116
ypT classification								
0	1.00 (0.78-1.29)	0.987	1.02 (0.79-1.33)	0.870	1.03 (0.80-1.33)	0.796	1.06 (0.81-1.38)	0.666
1-2	1.03 (0.85-1.27)	0.745	1.04 (0.84-1.27)	0.738	1.04 (0.85-1.28)	0.698	1.04 (0.84-1.29)	0.722

c. The relative risk between patient with or without adjuvant chemotherapy was calculated from Cox proportional hazard ratio model and adjusted for age, gender, cT/cN/ypT classification, surgery type, and CCI.

OS, overall survival; DFS, disease free survival; cT, clinical tumor; cN, clinical nodal; ypT, pathological tumor; CCI, Charlson Comorbidity Index.

**Table 3:** 5-year OS and DFS comparison by different clinicopathological stage and the use of adjuvant chemotherapy of study patients, n =720.

ypT classification	cT/cN classification	Without adjuvant chemotherapy N = 352			With adjuvant chemotherapy N = 368			*P*-value^d^	
		N	5- year OS (%)	5- year DFS (%)	N	5- year OS (%)	5- year DFS (%)	5- year OS	5- year DFS
ypT0N0	T3-4,N1-2	84	79 (94.05)	77 (91.67)	74	71 (95.95)	68 (91.89)	0.648	0.966
	T3-4, N0	37	34 (91.89)	32 (86.49)	28	23 (82.14)	23 (82.14)	0.356	0.766
	T1-2, N1-2	17	17 (100.00)	17 (100.00)	10	10 (100.00)	9 (90.00)	–	–
	T1-2, N0	17	14 (82.35)	14 (82.35)	3	3 (100.00)	3 (100.00)	0.477	0.477
ypT1-2N0	T3-4, N1-1	95	81 (85.26)	75 (78.95)	138	123 (89.13)	115 (83.33)	0.484	0.509
	T3-4, N0	58	46 (79.31)	45 (77.59)	66	59 (89.39)	55 (83.33)	0.123	0.411
	T1-2, N1-2	16	13 (81.25)	13 (81.25)	24	22 (91.67)	21 (87.50)	0.266	0.595
	T1-2, N0	28	26 (92.86)	22 (78.57)	25	17 (68.00)	16 (64.00)	0.030	0.317

d. P-value was derived from the Log rank test.

OS, overall survival; DFS, disease free survival; cT, clinical tumor; cN, clinical nodal; ypT, pathological tumor.

**Table 4:** The distribution and 5-year survival among different adjuvant chemotherapy regimens groups, n =368.

Drug name	Number of patientN (%)	5- year OS N (%)	5- year DFS N (%)
5-FU	210 (57.07)	188 (89.52)	173 (82.38)
Capecitabine	53 (14.40)	42 (79.25)	32 (60.38)
Oxaliplatin	64 (17.39)	52 (81.25)	43 (67.19)
Leucovorin	297 (80.71)	266 (89.56)	250 (84.18)
UFUR	161 (43.75)	137 (85.09)	125 (77.64)
Combined			
L+F	210 (57.07)	188 (89.52)	173 (82.38)
FOLFOX	58 (15.76)	46 (79.31)	37 (63.79)
CAPEOX	20 (5.43)	15 (75.00)	9 (45.00)
F+O	58 (15.76)	46 (79.31)	37 (63.79)
Monotherapy,			
No	326 (88.59)	289 (88.65)	273 (83.74)
Yes	42 (11.41)	39 (92.86)	37 (88.10)

OS, overall survival; DFS, disease free survival; L+F, Leucovorin+5-FU; FOLFOX, Oxaliplatin+ Leucovorin+5-FU; CAPEOX, Oxaliplatin + Capecitabine; F+O, Oxaliplatin+ 5-FU.

## Discussion

Treatment for clinical stage II or III rectal cancer patients consists of nCRT followed by total mesorectal excision and adjuvant chemotherapy with fluorouracil and oxaliplatin. However, the benefit of adjuvant chemotherapy for downstaged or good response (ypT0-2N0) rectal cancer after nCRT remains inconsistent (12–14). In this nationwide, population-based, cohort study, our results found no significant difference in 5-year OS or DFS between those who received adjuvant chemotherapy and those who did not. Moreover, there was no significantly difference in 5-year survival even in those with advanced cT or cN classification.

Our study offers a number of advantages over earlier studies from single institutions or several national datasets. First, we obtained data from the TCR and the NHIRD (18). This database covered more than 95% cancer cases in Taiwan. The follow-up period was long and the patient number was enough (N=764) to make our results convincing. Additionally, we included individuals with rectal cancer diagnosed between 2007 and 2018, which increased the relevance of our study. Second, this database had comprehensive information on patient characteristics, clinical staging, pathological staging, surgical methods and comorbidities enabling us to conduct in-depth analyses on the actual impact of adjuvant chemotherapy.

Adjuvant chemotherapy is recommended as standard treatment for those with high risk stage II and stage III colon cancer (19, 20). However, the precise benefit of adjuvant chemotherapy in rectal cancer remains unclear. According to NCCN guidelines, whether or not to give adjuvant chemotherapy depends on the pre-treatment clinical staging. The guidelines suggest all patients who underwent nCRT for locally advanced (T3/4 or node-positive) non-metastatic rectal cancer receive four months of adjuvant chemotherapy, regardless of the pathologic findings at the time of resection. However, in terms of the evidence level, four randomized phase III trials explored the benefit of adjuvant chemotherapy following nCRT for rectal cancer (6, 11, 21–23). None of the four found any advantage in the use of adjuvant chemotherapy, either in terms of recurrence rate or OS. However, all of these trials were flawed. For example, in the European Organisation for Research and Treatment of Cancer (EORTC) trial 22921 and the cooperative Italian study, the adjuvant chemotherapy regimen consisted of four to six cycles of postoperative bolus fluorouracil plus leucovorin, which was not consistent with the current standard regimen (24, 25). In these two studies, the rate of adherence to adjuvant chemotherapy was poor (43% in the EORTC 22921 study and 72% in the cooperative Italian study). In the Dutch colorectal PROCTOR/SCRIPT trials, the adjuvant chemotherapy regimens included fluorouracil/leucovorin or capecitabine (21). However, the trial did not reach full accrual. The adjuvant chemotherapy regimen in the United Kingdom phase III Chronicle trial was capecitabine plus oxaliplatin (XELOX), which was the current standard chemotherapy regimen (22). Unfortunately, the study was also closed prematurely due to poor accrual. A meta-analysis of individual patient data from all four of these trials concluded that fluorouracil-based chemotherapy did not improve OS, DFS, or distant recurrence rates (26). Another systematic review published in 2017 identified eight phase III trials and one randomized phase II trial comparing adjuvant chemotherapy with observation in patients with non-metastatic rectal cancer treated with nCRT. The authors reported that the data were not robust enough to warrant routine use of adjuvant therapy in this population (27). However, other meta-analyses have come to the opposite conclusion. A systematic review of the scientific literature from 1975 until March 2011 quantitatively summarized the available evidence regarding the impact of adjuvant chemotherapy on the survival of patients with surgically resectable rectal cancer (28). The authors supported the use of 5-FU based postoperative adjuvant chemotherapy, but available data did not allow them to define whether the efficacy of this treatment was greatest for one specific TNM stage. Consequently, conclusive data on the benefits of adjuvant therapy in rectal cancer patients remains lacking.

Our study focused on the benefit of adjuvant chemotherapy for patients with good response (ypT0-2N0) to nCRT and surgery. Patients who achieve a pathologic complete response after preoperative therapy have excellent outcomes (10). These findings raise concerns regarding the possibility of overtreatment in this group, when adding adjuvant chemotherapy. Other database studies have also failed to discover a significant benefit to adjuvant chemotherapy in this setting (29–31). However, three similar observational studies using the National Cancer Database, which looked only at patients achieving pathologic complete response, found that adjuvant chemotherapy did improve survival in this favorable subgroup (32–34).

Our study demonstrated that adjuvant chemotherapy is not beneficial for those rectal patients with good response to nCRT and surgery, even those with advanced clinical stage disease. However, our study showed that patients with clinical nodal positive status had worse OS and DSS. This finding implies that clinical stage might be a prognostic factor rather than a predictive factor. There are some potential explanations for the conflicting results, which are also the common limitations of nationwide cancer registry database analysis, including ours. First, it is challenging to accurately assess clinical stage, since the imaging tools used to determine stage differ by hospital. Some patients could be over-staged, which makes adjuvant chemotherapy appear to be of no benefit in this group. Second, the presence of perineural and extramural venous invasion, particularly after preoperative irradiation, is a significant negative prognostic factor for local recurrence, metastatic disease, and OS (35). However, this information was not recorded in our database. Third, due to treatment-related toxicity, patients receiving adjuvant chemotherapy may face interruption or dose reduction during the course of treatment. However, we could not assess the compliance with the administration of adjuvant chemotherapy (including prescribed dose, and cycles) (36, 37). Future work including the information about the compliance with the administration of chemotherapy may help us to investigate in depth about the role of adjuvant chemotherapy on survival. However, we could know the stop of neoadjuvant CRT course from the coding of total radiation dose. We found 20 (2.7%) patients received less than 45Gy during their neoadjuvant CRT. Fourth, patients with different molecular profiles, such as microsatellite instability, KRAS mutation, or BRAF mutation, may vary in their prognostic profiles and sensitivity to chemotherapeutic and biological agents. The microsatellite instability status also influences the decision to provide adjuvant chemotherapy, but these molecular profiles were not available for our analysis (38). Finally, following nCRT and surgery, patients with a pathologic complete response or a clinically significant downstage to ypT1/T2N0 often have a good prognosis and are unlikely to benefit from additional adjuvant chemotherapy. Although no markers were available in our cancer registry database, we defined the good response from the pathological stage (yp) which is generally accepted and used. However, the response should be a dynamic process and is better evaluated by tumor regression grade (39).

Questions remain regarding how much downstaging is predictive of further benefit from adjuvant therapy. It is possible that adjuvant chemotherapy may not benefit patients at the two extremes of pathologic response: patients with good response and those with poor or minimal pathologic response to nCRT. Perhaps the intermediate group may benefit from further adjuvant chemotherapy. However, identifying this group remains challenging and more prospective studies are needed before this occurs.

## Conclusion

In summary, our results demonstrated that adjuvant chemotherapy does not improve 5-year OS or DFS in rectal cancer patients with good response after nCRT and surgery. Moreover, there is no significantly difference in 5-year OS, even in those with advanced cT or cN classification. Therefore, adjuvant chemotherapy may be omitted in these good response patients. Prospective studies that include more patients and clinicopathological variables are necessary to valid our findings into clinical practice.

## Data availability statement

The original contributions presented in the study are included in the article/**Supplementary Material**. Further inquiries can be directed to the corresponding author.

## Author contributions

Study design: Y-HK, Y-TL, C-HH, C-LC, L-CC, C-JT, W-JH, Y-CC, C-CY. Data analysis: Y-HK, C-HH, Y-CC, C-CY. Manuscript writing: Y-HK, Y-TL, C-HH, C-CY. All authors contributed to the article and approved the submitted version.
