# Supplementary material for: Adjuvant chemotherapy and survival outcomes in rectal cancer patients with good response (ypT0-2N0) after neoadjuvant chemoradiotherapy and surgery: A retrospective nationwide analysis
Source: Front Oncol. 2022 Dec 16;12:1087778. doi: 10.3389/fonc.2022.1087778 (PMC9800595; doi:10.3389/fonc.2022.1087778)
Supplement: Supplementary file 1 [file Table_1.docx]

**Supplementary Table 1.** The information of neoadjuvant chemoradiotherapy of rectal cancer patients with good response (ypT0-2N0)

| **Variable** | **Adjuvant chemotherapy** | |
| --- | --- | --- |
|  | **Without** | **With** |
| **Overall** | **N=352** | **N=368** |
| **RT treatment time, days** |  |  |
| Median (Q1-Q3) | 37 (34-39) | 37 (34-39) |
| **Number of Fractions** |  |  |
| Median (Q1-Q3) | 26 (25-28) | 28 (25-28) |
| **RT dose, Gy** |  |  |
| Median (Q1-Q3) | 50.4 (50.0-50.4) | 50.4 (50.0-50.4) |
| <45, % | 10 (2.85) | 10 (2.72) |
| **Chemotherapy regimens** |  |  |
| 5-FU | 158 (44.89) | 207 (56.25) |
| Capecitabine | 61 (17.33) | 54 (14.67) |
| Oxaliplatin | 12 (3.41) | 18 (4.89) |
| Leucovorin | 228 (64.77) | 175 (47.55) |
| UFUR | 140 (39.77) | 126 (34.24) |
